# Model selection for metabolomics: predicting diagnosis of coronary artery disease using automated machine learning

**DOI:** 10.1093/bioinformatics/btz796

**Published:** 2019-11-08

**Authors:** Alena Orlenko, Daniel Kofink, Leo-Pekka Lyytikäinen, Kjell Nikus, Pashupati Mishra, Pekka Kuukasjärvi, Pekka J Karhunen, Mika Kähönen, Jari O Laurikka, Terho Lehtimäki, Folkert W Asselbergs, Jason H Moore

**Affiliations:** Department of Biostatistics, Epidemiology and Informatics, Institute for Biomedical Informatics, University of Pennsylvania, Philadelphia, PA, USA; Department of Cardiology, Division Heart and Lungs, Utrecht, The Netherlands; Department of Cardiology, Division Heart and Lungs, Utrecht, The Netherlands; Department of Clinical Chemistry, Fimlab Laboratories, Tampere, Finland; Department of Cardiology, Tampere University Hospital, Tampere, Finland; Department of Clinical Chemistry, Fimlab Laboratories, Tampere, Finland; Department of Cardiology, Tampere University Hospital, Tampere, Finland; Department of Cardio-Thoracic Surgery, Heart Center, Tampere University Hospital, Tampere, Finland; Department of Forensic Medicine, Fimlab Laboratories, Tampere, Finland; Department of Clinical Physiology, Tampere University Hospital, Tampere, Finland; Department of Cardio-Thoracic Surgery, Heart Center, Tampere University Hospital, Tampere, Finland; Department of Clinical Chemistry, Fimlab Laboratories, Tampere, Finland; Department of Cardiology, Tampere University Hospital, Tampere, Finland; Department of Cardiology, Division Heart and Lungs, Utrecht, The Netherlands; Health Data Research UK London, Institute for Health Informatics, University College London, London, UK; Institute of Cardiovascular Science, Faculty of Population Health Sciences, University College London, London, UK; Department of Biostatistics, Epidemiology and Informatics, Institute for Biomedical Informatics, University of Pennsylvania, Philadelphia, PA, USA

## Abstract

**Motivation:**

Selecting the optimal machine learning (ML) model for a given dataset is often challenging. Automated ML (AutoML) has emerged as a powerful tool for enabling the automatic selection of ML methods and parameter settings for the prediction of biomedical endpoints. Here, we apply the tree-based pipeline optimization tool (TPOT) to predict angiographic diagnoses of coronary artery disease (CAD). With TPOT, ML models are represented as expression trees and optimal pipelines discovered using a stochastic search method called genetic programing. We provide some guidelines for TPOT-based ML pipeline selection and optimization-based on various clinical phenotypes and high-throughput metabolic profiles in the Angiography and Genes Study (ANGES).

**Results:**

We analyzed nuclear magnetic resonance-derived lipoprotein and metabolite profiles in the ANGES cohort with a goal to identify the role of non-obstructive CAD patients in CAD diagnostics. We performed a comparative analysis of TPOT-generated ML pipelines with selected ML classifiers, optimized with a grid search approach, applied to two phenotypic CAD profiles. As a result, TPOT-generated ML pipelines that outperformed grid search optimized models across multiple performance metrics including balanced accuracy and area under the precision-recall curve. With the selected models, we demonstrated that the phenotypic profile that distinguishes non-obstructive CAD patients from no CAD patients is associated with higher precision, suggesting a discrepancy in the underlying processes between these phenotypes.

**Availability and implementation:**

TPOT is freely available via http://epistasislab.github.io/tpot/.

**Supplementary information:**

Supplementary data are available at *Bioinformatics* online.

## 1 Introduction

Although predictive analysis in biomedical research is typically based on deriving quantitative measures of confidence through the creation and fitting of a hypothesis-specific probability model, machine learning (ML)-based algorithms offers a wide range of different techniques that focus on prediction, through pattern recognition learning, with minimal underlying assumptions about the features. ML is especially effective when features are involved in nonlinear interactions or when no strong scientific hypothesis about feature interactions is established. In clinical research, ML-derived predictive models could facilitate preliminary hypothesis generation as well as be used for biomarker discovery through the selection of informative features. The choice of the most appropriate ML algorithm is a challenging process. Indeed, dozens of ML algorithms have been implemented in various languages: e.g. Scikit-learn library for Python (Pedregosa *et al.*, 2011), Weka software for Java (Witten *et al.*, 2016) and the caret package for R (Kuhn *et al.*, 2008). Each classification or regression ML algorithm contains numerous parameters that need to be selected and optimized. A common approach is to perform an exhaustive search over the selected algorithm parameter set (Bzdok *et al.*, 2018). Additionally, uncertainty in ML model selection comes from the number of various pre-processing algorithms such as, feature selectors (group of computational algorithms providing a reduction in the feature list according to a select statistical scoring metrics e.g. variance, f-value, χ^2^ etc.) and feature transformers [group of computational algorithms which provides transformation of the dataset with feature pre-processing (such as standardization and normalization), reduction of dimensionality of the feature set, or generation of new feature(s) from existing ones] that might be needed to enrich the data for signal. Together with ML model hyperparameter tuning, this creates numerous possible combinations to be validated on one particular dataset of interest. Automated ML (AutoML) seeks to take the guesswork out of this process by treating ML algorithms and pre-processing methods as building blocks for pipelines that are constructed and evaluated using a search algorithm.

AutoML methods to date have employed multiple optimization techniques: ML algorithm hyperparameter tuning implemented in the mlr R package (Bischl *et al.*, 2016), full pipeline Bayesian hyperparameter optimization used in Auto-WEKA (Thornton *et al.*, 2013) and auto-sklearn (Feurer *et al.*, 2015), Bayesian optimization of pipeline operators including the choice of imputer (group of algorithms providing a replacement of missing data with substituted values), selected feature transformers, ML model and calibrator is available via AutoPrognosis (Alaa and van der Schaar, 2018). Our specific AutoML method of interest is the tree-based pipeline optimization tool (TPOT) that takes a higher-level approach to the optimization process by using genetic programing to find optimal ML pipelines. TPOT has been observed to automatically generate ML pipelines that match or exceed the performance of traditionally tuned supervised ML algorithms (Olson and Moore, 2016). In clinical applications (Orlenko *et al.*, 2018), TPOT has delivered promising predictive performance while remaining robust to mixed datatypes and large feature spaces, containing clinical, demographic and biomarker data. Here we use TPOT to predict angiographic diagnosis of coronary artery disease (CAD) in the Angiography and Genes Study (ANGES) using metabolomics data. In addition, we provide a guideline for TPOT-based ML pipeline selection based on various clinical phenotypes and high-throughput metabolic profiles.

## 2 Materials and methods

### 2.1 TPOT overview

**Fig. 1.**
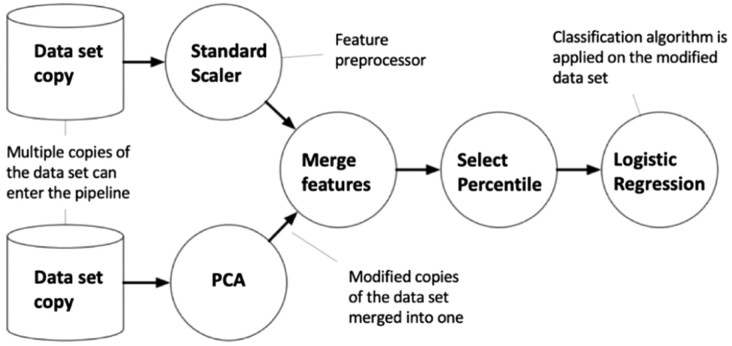
Example of the TPOT pipeline

Here we used TPOT as our AutoML method to generate optimized ML pipelines for the ANGES dataset. Briefly, TPOT employs genetic programing (Koza, 1992) from the Python package DEAP (Fortin *et al.*, 2012) to select series of data pre-processing functions and ML classification or regression algorithms that aim to maximize the performance of the model for a dataset of interest. In addition to ML algorithm, TPOT model pipeline (Fig. 1) may contain a diverse combinations of data transformers implemented in Scikit-learn Python library such as various types of pre-processors [Standard Scaler (SS), Min Max Scaler, Max Abs Scaler, Binarizer, Normalizer, polynomial features expansion] as well as various feature selectors [Variance Threshold, Select Percentile (SP), recursive feature elimination (RFE) etc.]. In certain cases, constructing a new feature set can be useful for extracting important information (e.g. when a selected method analyzes one feature at a time while complex feature interactions are present in the dataset). TPOT also contains several custom feature constructor implementations: zero counts (count of zero/non-zeros per sample), stacking estimator (SE) (generates predictions and class probabilities with a classifier of choice as new features), one hot encoder (transforms categorical feature into binary features) and a selection of sklearn transformer implementations: PCA, independent component analysis, non-linear transformations through kernel approximation (Nystroem, RBF Sampler). The TPOT full configuration consists of 11 classification algorithms, 14 feature transformers and 5 feature selectors (for the full list please refer to the TPOT website). To combine all of these operators, TPOT employs a tree-based structure (Fig. 1 and   SupplementaryFig. S1): every pipeline starts with one or more copies of the entire dataset at the beginning of the tree structure and proceeds with feature transformation/selection operators described above or ML algorithm. Operators then modify the original dataset and it is further passed along the tree to the next operator or in the case if there are multiple copies of the dataset it may be combined into a single set via a combination operator.

The automatic optimization process via genetic programing begins with the initialization of a population of pipelines by randomly generating a fixed number of tree-based pipelines that is further subjected to the evolutionary algorithm through rounds (generations) of mutation, recombination of pipeline components and selection. The fitness of the pipeline is calculated via Pareto multi-objective function that aims to maximize ML algorithm’s performance metrics of choice (accuracy, precision, recall, f1 score, *r*^2^, mean squared error etc.) while minimizing the pipeline’s complexity (i.e. the number of data transformers/selectors in the pipeline). There are several sources of variation that changes the structure of a TPOT pipeline and facilitates the selection of the most fit for a given dataset: crossover and mutation. First, one-point crossover applied to a user-specified percentage of pipelines where two randomly selected pipelines split at random point in the tree and their contents exchanged. Subsequently, mutation is applied at a fixed user-defined frequency with changes in the form of addition, removal or substitution of the pipeline operators. Once the changes have been introduced, and their fitness effect calculated, the TPOT pipeline with the highest fitness from the current generation is selected to replace 10% of the population in the next generation. The remaining 90% of the population is selected via three-way tournament with two-way parsimony: three pipelines selected for the tournament where at first the lowest fitness one is removed and then the least complexed of the remaining two is selected to be reproduced in the next generation. Population size, generation number and mutation rate parameters could be defined by the user. Here we will be comparing TPOT-based model optimization to the more traditional exhaustive tuning of the selected ML algorithms parameters.

### 2.2 TPOT model selection for CAD phenotype in ANGES

#### 2.2.1 Study population

This study is based on data from the ANGES, which enrolled 1000 patients referred to coronary angiography at Tampere University Hospital (Finland) between September 2002 and July 2005 (for details see Mennander *et al.*, 2008). This study included 925 patients from whom coronary angiography results and serum samples for metabolic profiling were available. Patients were categorized into three groups according to the angiographic findings. Obstructive or functionally relevant CAD was defined as stenosis of ≥50% stenosis of any major coronary artery (left anterior descending, left circumflex or right coronary artery). Less than 50% coronary artery stenosis was categorized as a non-obstructive CAD. Patients were considered to have no CAD if no major coronary artery showed any sign of stenosis. ANGES approved by the Ethics Committee of Tampere University Hospital. All patients gave written informed consent, and the study conforms to the Declaration of Helsinki.

#### 2.2.2 Metabolic profiling

Fasting EDTA serum samples were stored at −80°C prior to analysis. A high-throughput ^1^H NMR metabolomics platform (Soininen *et al.*, 2015) was used to quantify 73 lipid and metabolic measures: 56 lipid-related measures (including concentrations of 14 lipoprotein subclasses), 8 amino acids, 4 glycolysis-related metabolites and 5 other metabolites.

#### 2.2.3 Study design

In addition to 73 metabolic features, we included 27 demographic and clinical features. Baseline characteristics of the study population are listed in Supplementary Table S1. We processed the missing values with KNN imputation strategy using fancyimpute python package (Rubinsteyn *et al.*, 2016). We used TPOT in classification mode as our datasets of interest have binary phenotypes. In clinics, non-obstructive is distinguished from obstructive CAD based on the extent of the coronary stenosis to identify patients requiring revascularization, even though both CAD phenotypes represent the same pathophysiological pathway. We, therefore, performed comparisons for two different profiles: no CAD versus non-obstructive and obstructive CAD (P1) and no CAD and non-obstructive CAD versus obstructive CAD (P2). We split the datasets into training (75%) and validation (25%) sets and run all optimization approaches on the training set. We reported both training and validation sets scores in the result section.

TPOT was set to run for 1000 generations or 24 h (whichever happens first) with the population size of 1000 pipelines. For each phenotypic profile TPOT-based model selection was performed with different configuration: full configuration with complete list of data operators and ML classification models (Model 1), reduced configuration with logistic regression (LR) classifier and complete list of data transformers and selectors (Model 2), reduced configuration with decision tree (DT) classifier and complete list of data transformers and selectors (Model 3), reduced configuration with random forest (RF) classifier and complete list of data transformers and selectors (Model 4). The performance of each pipeline was estimated with 10-fold cross-validation and balanced accuracy. Balanced accuracy is a metric used in imbalanced datasets in order to avoid inflated performance estimates. It evaluates the accuracy of each class and then computes an unweighted average of each class accuracy. In the case of the balanced dataset it will be equal to the accuracy score (Mosley, 2010). Since evolutionary computation is a stochastic process, each configuration was run 50 times with different random seeds and the pipeline with the highest balanced accuracy score was selected as the representative model. This optimization strategy has been chosen empirically. We usually recommend running TPOT for 1000 generations which can take significant amount of time in cases of a large datasets. We propose using multiple random seeds in order to compensate for any potential effects from the initialization step.

We further compared TPOT-based model selection to the exhaustive grid search parameter tuning of LR classifier (Model 5), DT classifier (Model 6) and RF Classifier (Model 7). The choice of tree-based classifiers for evaluation, in addition to LR, is justified by the results of the previous benchmarking studies where tree-based methods reported superior performance in comparison to the other ML classifiers (Fernández-Delgado *et al.*, 2014; Olson *et al.*, 2018) Additionally, the grid search optimization method was applied to the ML classifiers from the TPOT-generated pipelines that reported the highest performance that were combined into a pipeline with a subset of data pre-processors. Since the penalty parameter depends on the scale of each feature, we standard-scaled (z-transformed) all features before training linear regression models with and without feature selection. We also applied standard scaling before training naïve Bayes classifiers for multivariate Bernoulli models. The naïve Bayes classifier for Bernoulli models implemented in sklearn performs binarization of the continuous features with a default threshold of 0.0. Prior standard scaling thus results in mean binarization of continuous features. We have reported different performance metrics (balanced accuracy, area under the curve (AUC), Precision-Recall curve (PRC), Precision, Recall) for all ML pipelines along with model complexity (the number of data transformation steps).

The predictive power of the clinical and metabolic features was reported for the best pipelines for both phenotypic profiles. To calculate the coefficients of the predictive ability of the specific feature we used permutation feature importance (PFI) approach. Within this approach we first calculated a pipeline performance (balanced accuracy) on the unchanged dataset, and then permuted the values within a feature and calculate the performance of the pipeline on the modified dataset. The resulting difference in performances is the PFI score. This procedure was repeated 100 times for each feature and the mean taken as a final PFI score.

## 3 Results

### 3.1 Model selection with TPOT

Table 1(A) outlines the summary of the comparative analysis of model selection from the TPOT optimization process and grid search parameter tuning for P1 phenotype. As a result of TPOT optimization with full configuration pipeline Model A1 has been selected with the validation set balanced accuracy 0.77. It contains four pre-processing operators (RFE, SE with LR Classifier, SE with Multinomial Naïve Bayes Classifier, SS and Bernoulli Naïve Bayes (BNB) as ML classifier). TPOT optimization with reduced configuration of LR-only classifier (Model A2) has produced a pipeline with eight pre-processors (SE with LR Classifier, Zero Counts, Select From Model with Extra Trees Classifier, Binarizer, Normalizer and SS) and validation set balanced accuracy 0.76. Model A3 is the result of TPOT reduced configuration with DT Classifier only had a pipeline with six pre-processors [Binarizer, Select Few, SE with DT Classifier^1^, Normalizer^1^, SE with DT Classifier^2^ and Normalizer^2^ (here and further abbreviation^1^ and ^2^identify that operator was used twice within the pipeline but with a different hyperparameters setup)] with the validation set balanced accuracy 0.70. Model A4 has been selected during TPOT RF Classifier only optimization and had three pre-processors (SE with RF Classifier and Normalizer) along with grid search parameter tuning for LR, DT and RF classifiers (Models A5–A7 correspondingly) reported noticeably lower validation set balanced accuracy performance (balanced accuracy 0.61–0.69). Overall, the first two models have reported very similar performances in comparison to the remaining models for the P1; however, ultimately the best model appears to be Model A1 selected by the TPOT optimization with full configuration.

**Table 1.** Comparative analysis of the TPOT optimization of selected model with various performance metrics for P1(A) and P2(B).

Model	Balanced accuracy V/T	Precision V/T	Recall V/T	ROC AUC V/T	PRC V/T	Pipeline complexity
A. P1						
A1. TPOT (BNB)	**0.77/0.79**	**0.91/0.93**	0.77/0.79	**0.77/0.86**	**0.88/0.95**	5
A2. LR TPOT	0.76/0.79	0.90/0.93	0.79/0.80	0.76/0.86	0.87/0.95	9
A3. DT TPOT	0.70/0.74	0.88/0.89	0.71/0.80	0.70/0.74	0.85/0.87	7
A4. RF TPOT	0.69 /0.69	0.85/0.86	0.85/0.88	0.61/0.81	0.81/0.94	4
A5. LR GS	0.68/0.72	0.85/0.87	0.85/0.90	0.68/0.87	0.83/0.95	1
A6. DT GS	0.61/0.67	0.81/0.83	0.86/0.84	0.61/0.72	0.81/0.87	1
A7. RF GS	0.61/0.66	0.81/0.84	**0.87/0.88**	0.64/0.81	0.82/0.93	1
B. P2						
B1. TPOT (BNB)	**0.78/0.78**	**0.82/0.84**	0.79/0.79	**0.78/0.82**	**0.78/0.86**	5
B2. LR TPOT	0.77/0.75	0.80/0.80	0.84/0.80	0.77/0.84	0.76/0.90	5
B3. DT TPOT	0.75/0.76	0.78/0.81	0.84/0.81	0.72/0.80	0.73/0.84	6
B4. RF TPOT	0.76/0.76	0.78/0.81	**0.86/0.83**	0.75/0.83	0.74/0.88	4
B5. LR GS	0.73/0.74	0.76/0.78	0.84/0.84	0.73/0.85	0.73/0.89	1
B6. DT GS	0.74/0.73	0.78/0.80	0.81/0.76	0.74/0.78	0.74/0.83	1
B7. RF GS	0.72/0.72	0.77/0.78	0.76/0.81	0.69/0.83	0.70/0.88	1

***Note***: Metrics’ score are shown for validation (V) and training (T) set. The highest score in each metrics category is marked via bold font. The best model for each phenotypic profile was selected according to the highest balanced accuracy. BNB, Bernoulli Naïve Bayes classifier; LR, logistic regression classifier; DT, decision tree classifier; RF, random forest classifier; GS, grid search optimization.

Similarly, we have summarized models selected for P2 phenotypes via Table 1(B). TPOT with the full configuration produced pipeline with four pre-processors (Variance Threshold, RFE, SE with LR, Max Abs Scaler) and BNB (Model B1) with the validation set balanced accuracy 0.78. TPOT with the reduced configurations have produced slightly less accurate models with validation set balanced accuracy 0.77 for Model B2 or LR Classifier also with four pre-processors (RFE^1^, SE with LR Classifier, SS and RFE^2^), balanced accuracy 0.75 for Model B3 or DT Classifier with five pre-processors (Binarizer, SE with DT Classifier, Select Fwe, Nystroem and PCA), balanced accuracy 0.76 for Model B4 or RF Classifier with three pre-processors (Binarizer, Normalizer and SP). Grid search tuning of LR, DT and RF classifiers produced balanced accuracy in the range 0.70–0.74. Overall, the best predictive performance came from the TPOT full configuration selection (Model B1).

**Fig. 2.**
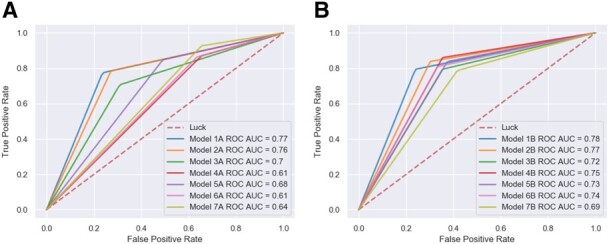
ROC AUC curves for selected models for P1 dataset (**A**) and P2 dataset (**B**)

**Fig. 3.**
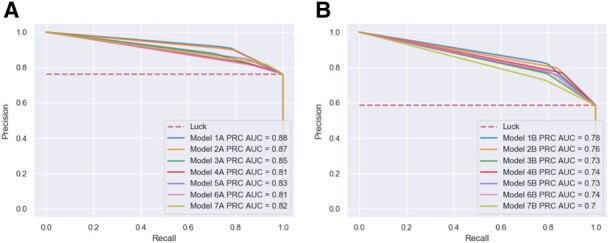
Precision-recall curves for selected models for P1 dataset (**A**) and P2 dataset (**B**)

In ML binary classification analysis, current research has shifted away from simply presenting the accuracy of the model when reporting clinically relevant findings. Therefore, in this study, predictive power of the selected models was additionally evaluated via precision, recall, and threshold-based performance metrics. One of the most common metrics employed in clinical settings is precision or positive predictive values that characterizes the model’s ability to not to label negative samples as positive samples. It is often complemented by recall metrics—the ability of the model to detect all the positive samples. These metrics are single-threshold, meaning that they are defined for a single choice of decision threshold for an ML classifier and therefore are not able to describe its behavior within a range of decision criterions. This problem, however, could be resolved by reporting various receiver operator characteristic (ROC) curves (Provost *et al.*, 1998). The most commonly used one, ROC area AUC, illustrates the results of binary classification problem as classifier threshold is varied. It reports how the true positive rate (number of correctly classified positive samples) varies with the false positive rate (number of incorrectly classified negative samples; Fig. 2). Models with BNB classifier optimized by TPOT reported the top ROC score for both P1 and P2. Similar to ROC AUC, PRC plots provides classifier-wide estimation; however, when dealing with imbalanced dataset PRC provides a more informative description of the classifier performance (Davis and Goadrich, 2006; Saito and Rehmsmeier, 2015). According to the PRC curves (Fig. 3), TPOT models with BNB classifier (Models A1 and B1) have reported the top performance for both P1 and P2 similar to the other metrics. Overall, BNB classifier models optimized by TPOT have been ultimately first among four out five performance metrics reported here for both P1 and P2.

### 3.2 Complexity and performance tradeoff in model selection

We further raised the question about the relationship between a model’s complexity and its performance with our assumption that a more complex model generally performs better than a less complex model. Indeed, for both P1 and P2 the best performing models (Models A1 and B1) had a complexity of five, where, in addition to optimized ML algorithm, four data transforming operators had been selected. To test the robustness of this model, we performed a sensitivity analysis where we consecutively removed pre-processing operators from the model pipeline to track the changes in its performance. As it can be seen from the Table 2(A), the balanced accuracy and ROC AUC performance dramatically decrease after removal of the first two pre-processors (RFE and SE with LR Classifier). This suggests that this particular dataset may contain intricate non-linear relationships among features so high complexity combination of data transformers is needed to describe these relationships. Similar situations were observed for the model selected for P2: removal of the first three pre-processors (Variance Threshold, RFE, SE with LR) led to 10% decrease in both balanced accuracy and ROC AUC metrics [Table 2(B)]. In summary, we observed a consistent decrease across all performance metrics over the decrease in complexity confirming the general trend for increased complexity—increased performance relationship.

**Table 2.** The complexity-performance relationship for models selected by the TPOT optimization for P1(A) and P2(B)

Model	Balanced accuracy V/T	Precision V/T	Recall V/T	ROC AUC V/T	PRC V/T	Pipeline complexity
A. P1						
Model A1	0.77/0.79	0.91/0.93	0.77/0.79	0.77/0.86	0.88/0.95	5
Pr-1	0.74/0.77	0.90/0.92	0.77/0.79	0.74/0.84	0.86/0.94	4
Pr-2	0.67/0.73	0.86/0.90	0.73/0.72	0.67/0.80	0.83/0.92	3
Pr-3	0.64/0.69	0.84/0.89	0.7/0.68	0.64/0.76	0.82/0.91	2
Pr-4	0.62/0.61	0.81/0.81	0.95/0.96	0.62/0.85	0.81/0.95	1
B. P2						
Model B1	0.78/0.78	0.82/0.84	0.79/0.79	0.78/0.82	0.78/0.86	5
Pr-1	0.78/0.76	0.82/0.82	0.81/0.79	0.78/0.81	0.78/0.86	4
Pr-2	0.74/0.76	0.79/0.82	0.77/0.78	0.74/0.81	0.74/0.86	3
Pr-3	0.68/0.75	0.75/0.82	0.7/0.75	0.68/0.8	0.7/0.85	2
Pr-4	0.68/0.75	0.75/0.82	0.7/0.75	0.68/0.8	0.7/0.85	1

***Note*** *:* Model ‘Pr-1’, ‘Pr-2’ etc. indicate the number of pre-processors removed from the original model pipeline.

Given the results of the model selection process for P1, we concluded that a proper choice and optimization of data pre-processing operators and ML algorithms could be equally important if the goal is to obtain a maximum performance score. Therefore, to make a fair comparison of the TPOT optimization with competitive grid search-based model selection approach we have evaluated the performance of LR and BNB ML algorithms in various combinations with SS, SP and RFE pre-processors (Table 3). For P1 addition of SS operator in combination with RFE selector to the LR algorithm resulted into noticeable increase in balanced accuracy (from 0.68 to 0.73). Further addition of feature selectors, however, only added 1% increase in balanced accuracy score for both profiles.

**Table 3.** Comparative analysis of the grid search optimization of selected ML algorithms with SS, SP and RFE pre-processing operators for P1(A) and P2(B)

Model	Balanced accuracy V/T	Precision V/T	Recall V/T	ROC AUC V/T	PRC V/T	Pipeline complexity
A. P1						
LR pipelines						
LR	0.68/0.72	0.85/0.87	0.85/0.90	0.68/0.87	0.84/0.95	1
LR +SS	0.68/0.77	0.86/0.92	0.76/0.80	0.68/0.84	0.84/0.94	2
LR + SS + SP	0.68/0.78	0.86/0.92	0.76/0.81	0.68/0.84	0.84/0.94	3
LR + SS + RFE	0.73/0.77	0.88/0.91	0.80/0.81	0.73/0.84	0.86/0.94	3
BNB pipelines						
BNB	0.72/0.77	0.88/0.91	0.76/0.78	0.72/0.85	0.85/0.95	1
BNB + SS	0.66/0.72	0.85/0.89	0.73/0.73	0.66/0.79	0.83/0.92	2
BNB + SS + SP	0.71/0.76	0.88/0.92	0.76/0.75	0.71/0.84	0.85/0.95	3
BNB + SS + RFE	0.70/0.73	0.88/0.90	0.75/0.76	0.70/0.82	0.85/0.94	3
B. P2						
LR pipelines						
LR	0.73/0.74	0.76/0.78	0.84/0.84	0.73/0.85	0.73/0.89	1
LR +SS	0.69/0.76	0.73/0.81	0.79/0.80	0.69/0.84	0.70/0.89	2
LR + SS + SP	0.72/0.76	0.76/0.81	0.80/0.79	0.72/0.83	0.73/0.89	3
LR + SS + RFE	0.74/0.74	0.78/0.80	0.81/0.80	0.74/0.84	0.74/0.89	3
BNB pipelines						
BNB	0.70/0.74	0.75/0.80	0.77/0.79	0.70/0.80	0.71/0.85	1
BNB + SS	0.63/0.66	0.69/0.75	0.69/0.67	0.63/0.74	0.66/0.81	2
BNB + SS + SP	0.74/0.75	0.80/0.83	0.74/0.75	0.74/0.82	0.74/0.87	3
BNB + SS + RFE	0.65/0.67	0.71/0.76	0.74/0.73	0.65/0.77	0.68/0.83	3

***Note*** *:* SS, standard scaler; SP, select percentile*;* RFE, recursive feature eliminator; LR, logistic regression classifier; BNB, Bernoulli Naïve Bayes classifier.

Addition of SS to BNB algorithm, on the contrary, reduced the model performance according to all reported performance metrics for both profiles. However, the further addition of SP feature selector to the pipeline resulted into an improvement of BNB pipeline performance according to balanced accuracy for P2 profile. This comparative analysis provides a brief insight into the importance of the correct choice and optimization of the data pre-processing operators or their combination for clinical datasets with complex relationships. Although the addition of specific pre-processors enhanced grid search optimization as compared with the ML algorithm tuning itself, TPOT agnostic optimization still provides with the best overall ML solution for both phenotypic profiles.

### 3.3 Feature importances

**Fig. 4.**
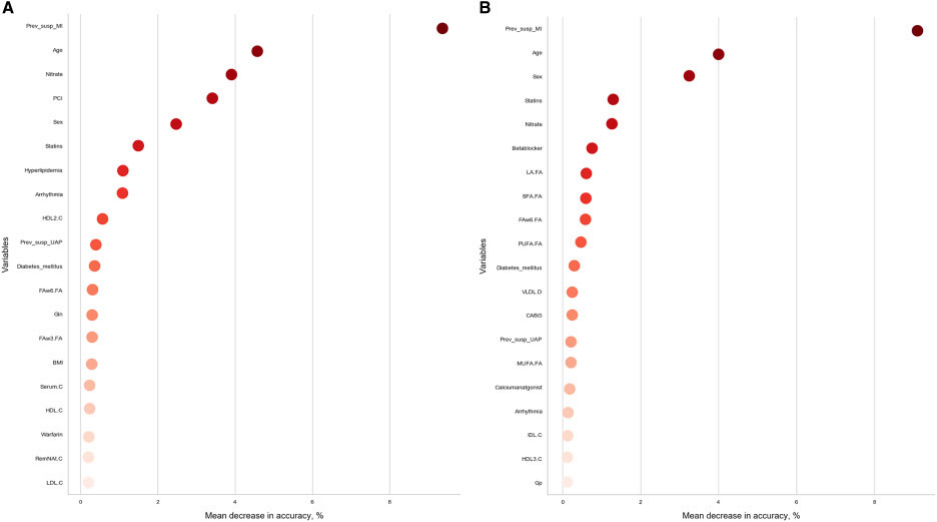
PFI coefficients produced by Model A1 for P1 dataset with validation set balanced accuracy 0.77 (**A**) and by Model B1 for P2 dataset with validation set balanced accuracy 0.78 (**B**)

The TPOT selection process generated ML model pipelines that outperformed the traditional exhaustive parameter search approach for both CAD phenotypic profiles. Within clinical settings, one of the most important considerations for an ML model is interpretation via importance coefficients for features. Some models provide built-in functions such as coefficients in linear regression or feature importance scores in tree-based models. However, it is often the case that ML algorithms don’t have an implemented feature importance function or a model pipeline could be too complex to use this function. In these cases, PFI can be a great alternative metric. Here we report 30 features with the top coefficients received from the PFI analysis performed on the P1 dataset with Model A1 and P2 dataset with Model B1 (Fig. 4A and B). The top coefficients correspond to clinical features such as sex, age, information about medication previously taken and several metabolic determinants like overall lipid content and HDL for P1 (Fig. 4A) and various fatty acids for P2 (Fig. 4B). Interestingly, both subsets report a rapid change in decline of the mean decrease in balanced accuracy coefficients after the top ten features, e.g. for P1 (Fig. 4A) coefficients for the top 10 features experience a decrease from 8.8 (Prev_susp_MI) to 0.88 (Arrhythmia) while the remaining top features slowly approach coefficient of zero and with that behavior create a threshold for selection of the most important features that could be used in CAD diagnosis predictions.

## 4 Discussion

In this study, we present a comprehensive analysis of AutoML methods implemented in TPOT to predict the angiographic diagnosis of CAD, using metabolic and clinical features. We demonstrate that for the ANGES dataset, the TPOT model selection approach produces classification pipelines that outperform exhaustive grid search optimization (Tables 1 and 3). As reported in the summary tables (Table 1), the BNB classifier in combination with four pre-processors showed the best performance for P1, with a validation set balanced accuracy 0.77. In comparison, the grid search optimized LR yielded a balanced accuracy 0.68 which generates a 9% difference. With a balanced accuracy 0.78, BNB classifier combined with four pre-processors was the best performing model for P2, which outperformed the best grid search optimized model—DT classifier by 4%.

The agnostic approach that uses minimum assumption about model selection employed by TPOT offers great potential for clinical prediction, particularly if the underlying mechanistic relationship between the different features is unknown. Here we employed two TPOT-based optimization approaches: full configuration with a complete list of pre-processors and classifiers and reduced configuration with a complete list of pre-processors and one selected classifier (LR, DT or RF). We have observed that in both instances TPOT with full set of classifiers and pre-processors had generated the model pipelines that outperform all optimization strategies (including reduced configuration TPOT and grid search with/without pre-processors). This suggests that the choice of pre-processors, and its combinations, could be as important as the choice of ML algorithm and its parameters (e.g. Table 2 shows that for P1 removing first 2 pre-processors decreases balanced accuracy on 10%). That was also demonstrated via comparison of grid search optimization with selected data pre-processors (Table 3). Alternatively, to perform the agnostic model selection via the grid search approach one will need extensive computational resources. For example, to optimize an ensemble tree-based ML algorithm with five hyperparameters with 10 possible values for each, grid search will need to sample 100 000 combinations to select the best one and it would take over 2700 computing hours (with 32 GB RAM Desktop, 10 s per combination). A list of pre-processors can significantly increase the running time: adding 10 pre-processors to the space search will increase the number of combinations beyond 9.8 trillion (or over 270 billion computing hours). This would be an estimate for the pre-processors with no hyperparameters. However, many contain at least one hyperparameter to tune which would greatly increase the total number of combinations. Therefore, an agnostic approach to model selection with grid search may not be feasible with modern computational resources and thus stochastic search methods such as genetic programing may be the best option.

As we mentioned earlier, the best classification performance was associated with full configuration TPOT optimization that resulted in pipelines with the BNB classification algorithm for both P1 and P2. This algorithm is rarely considered for classification task in biomedical predictive analytics and is most often used in spam detection procedures. The BNB classifier contains a binarization threshold function that could be relevant to some datasets, especially those with binary and/or discrete features. Indeed, the top features detected via PFI analysis were binary in both profiles. However, BNB classifier outperforms all the competitive algorithms only with the select pre-processors, with the most impactful ones to be RFE selector and SE with LR Classifier (Table 2). The first one uses Extra Tree Classifier estimation to reduce feature set in half, the second one generates class probabilities with the LR classifier as a synthetic feature, and therefore in addition to BNB assumptions this pipeline contains informational contribution from tree-based and linear models. Overall, making minimum assumption when building a model (and selecting feature transformers and selectors) could be very useful strategy when little is known about relationships between features and phenotype (e.g. for early hypothesis generation studies).

**Fig. 5.**
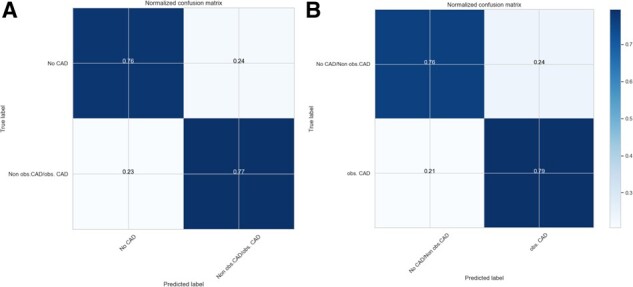
Normalized confusion matrix for selected TPOT-optimized models for P1 dataset (**A**) and P2 dataset (**B**)

We applied selected models to compare phenotypic profiles with a goal to clarify the role of non-obstructive CAD patients in CAD diagnostics. As a result of this comparison, the TPOT optimization produced a model pipeline with similar classification accuracy for both non-obstructive CAD/no CAD versus no CAD profile and obstructive CAD versus non-obstructive CAD/no CAD profile (BNB TPOT, 0.78) (Table 1 and Fig. 5) but closer look across all metrics identified a substantially higher precision and precision-recall curve values associated with the first profile (Table 1 and Fig. 2). Obstructive CAD is defined using arbitrary thresholds (in our study 50% stenosis) to identify patients requiring revascularization. However, non-obstructive and obstructive CAD represent the same pathophysiological pathways, complicating the identification of discriminative features. Therefore, we performed an examination of selected models with regard to the predictive power of the features via PFI procedure. As a result, a subset of top predictive clinical and metabolic features was outlined (Fig. 4) for both phenotypic profiles. For the P1 profile a number of known risk factors (Hajar, 2017) and phenotypic proxies were found among top features including myocadiac infarction signs, age, sex, history of percutaneous coronary intervention (PCI) procedure, administration of nitrate and statin medications, hyperlipidemia and HDL cholesterol. The top predictive features differ between P1 and P2: in the P2 dataset metabolic features and specifically fatty acids seems to be more relevant for the prediction of CAD diagnosis (linoleic acid, total saturated fatty acid, omega-6 fatty acid and polyunsaturated fatty acid cumulative coefficient >2%). These deviations could be related to non-obstructive CAD effect and the difference in the model pipeline selected for each profile. Overall, clinical information in combination with selected metabolic features (HDL, fatty acids) are potent predictors of CAD diagnosis.

## 5 Conclusion

We demonstrated the power of agnostic model selection with the AutoML tool TPOT for CAD diagnosis prediction using clinical and metabolic data from the ANGES cohort. As a result, TPOT optimization automatically produced predictive models that outperformed grid search optimized models. We used selected models to show that phenotypic profile that distinguishes non-obstructive CAD patients from no CAD patients is associated with higher precision and subsequently have a different subset of predictive features than phenotypic profile that treats no CAD patients and non-obstructive CAD patients as the same outcome.

## Authors’ contribution

A.O. and D.K. contributed to the conception of the project, conduct of the data analysis, and the interpretation of the results, and wrote the manuscript. F.W.A. and J.H.M. contributed to the conception of the project and the interpretation of the results and edited the manuscript for intellectual content. L.P.L., K.N., P.M., P.K., P.J.K., M.K., J.O.L. and T.L. contributed to the acquisition of the data and provided critical revisions to the manuscript.

## Funding

This work was supported by grant [R01 LM010098] from the National Institutes of Health (USA).

*Conflict of Interest*: none declared.

## Supplementary Material

btz796_Supplementary_Data
