# Supplementary material for: Model selection for metabolomics: predicting diagnosis of coronary artery disease using automated machine learning
Source: Bioinformatics. 2019 Nov 8;36(6):1772–8. doi: 10.1093/bioinformatics/btz796 (PMC7703753; doi:10.1093/bioinformatics/btz796)
Supplement: btz796_Supplementary_Data [file btz796_supplementary_data.zip › btz796-Suppl_Data/TableS3.docx]

Table S3. Running time of different optimization approaches for P1 (A) and P2 (B) datasets.

| **Model** | **Running time, s** |
| --- | --- |
| 1. **P1** | |
| **TPOT (full and reduced configurations)** | 86 400 |
| **LR** | 64 |
| **DT** | 2 687 |
| **RF** | 352 421 |
| **SS+LR** | 76 |
| **SS+SP+LR** | 3 323 |
| **SS+RFE+LR** | 106 612 |
| **BNB** | 0.61 |
| **SS+BNB** | 0.93 |
| **SS+SP+BNB** | 379 |
| **SS+RFE+BNB** | 59 052 |
| 1. **P2** | |
| **TPOT (full and reduced configurations)** | 86 400 |
| **LR** | 64 |
| **DT** | 2 880 |
| **RF** | 396 076 |
| **SS+LR** | 75 |
| **SS+SP+LR** | 3 000 |
| **SS+RFE+LR** | 124 646 |
| **BNB** | 0.61 |
| **SS+BNB** | 0.9 |
| **SS+SP+BNB** | 321 |
| **SS+RFE+BNB** | 70 469 |
