# Supplementary material for: Model selection for metabolomics: predicting diagnosis of coronary artery disease using automated machine learning
Source: Bioinformatics. 2019 Nov 8;36(6):1772–8. doi: 10.1093/bioinformatics/btz796 (PMC7703753; doi:10.1093/bioinformatics/btz796)

Figure S2. Boxplot/swarmplot depicting classification performances of replicates of various TPOT configurations.


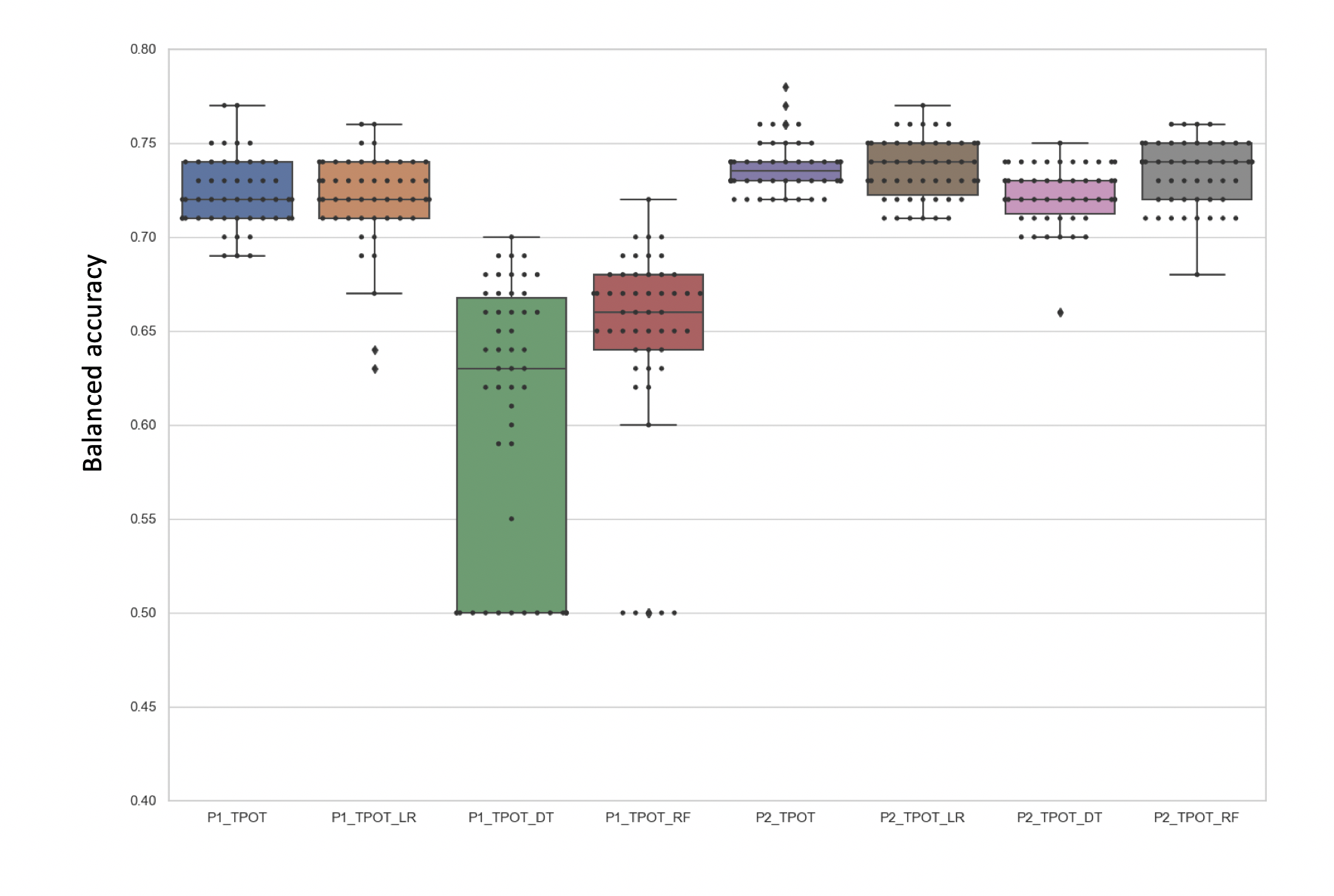

Supplement: btz796_Supplementary_Data [file btz796_supplementary_data.zip › btz796-Suppl_Data/FigureS2.docx]
