# Supplementary material for: Model selection for metabolomics: predicting diagnosis of coronary artery disease using automated machine learning
Source: Bioinformatics. 2019 Nov 8;36(6):1772–8. doi: 10.1093/bioinformatics/btz796 (PMC7703753; doi:10.1093/bioinformatics/btz796)
Supplement: btz796_Supplementary_Data [file btz796_supplementary_data.zip › btz796-Suppl_Data/TableS1.docx]

Table S1.Population description for P1 (A) and P2 (B)

| 1. P1 | | |
| --- | --- | --- |
|  | **No CAD**  **(n=214)** | **no obs. CAD/obs. CAD**  **(n=714)** |
| Age, years | 57.55 [51.42, 66.07] | 65.15 [57.40, 71.60] |
| Male sex | 88 (41.1) | 509 (71.3) |
| BMI, kg/m2 | 27.40 [24.70, 30.50] | 27.70 [25.10, 30.50] |
| Smoker | 27 (12.6) | 102 (14.3) |
| Diabetes mellitus | 36 (16.8) | 232 (32.5) |
| Hypertension | 120 (56.1) | 433 (60.6) |
| Arrhythmia | 60 (28.0) | 135 (18.9) |
| Hyperlipidemia | 125 (58.4) | 575 (80.5) |
| Family history CVD | 135 (63.1) | 485 (67.9) |
| Lipid measures |  |  |
| Total cholesterol, mmol/L | 4.47 [3.89, 5.12] | 4.17 [3.59, 4.82] |
| HDL-C, mmol/L | 1.38 [1.14, 1.63] | 1.19 [0.98, 1.41] |
| Triglycerides, mmol/L | 1.24 [0.89, 1.71] | 1.45 [1.07, 1.89] |
| Medication |  |  |
| Statins | 88 (41.1) | 527 (73.8) |
| Betablockers | 138 (64.5) | 603 (84.5) |
| ACE inhibitors | 43 (20.1) | 209 (29.3) |
| Diuretics | 53 (24.8) | 175 (24.5) |
| 1. P2 | | |
|  | **No CAD/**  **no obs. CAD**  **(n=370)** | **Obs. CAD**  **(n=558)** |
| Age, years | 58.95 [52.92, 67.55] | 65.55 [58.23, 72.10] |
| Male sex | 169 (45.7) | 428 (76.7) |
| BMI, kg/m2 | 27.40 [24.80, 30.67] | 27.85 [25.10, 30.40] |
| Smoker | 52 (14.1) | 77 (13.8) |
| Diabetes mellitus | 78 (21.1) | 190 (34.1) |
| Hypertension | 211 (57.0) | 342 (61.3) |
| Arrhythmia | 93 (25.1) | 102 (18.3) |
| Hyperlipidemia | 246 (66.5) | 454 (81.4) |
| Family history CVD | 239 (64.6) | 381 (68.3) |
| Lipid measures |  |  |
| Total cholesterol, mmol/L | 4.41 [3.83, 5.04] | 4.15 [3.55, 4.75] |
| HDL-C, mmol/L | 1.34 [1.06, 1.58] | 1.16 [0.98, 1.38] |
| Triglycerides, mmol/L | 1.29 [0.97, 1.80] | 1.47 [1.10, 1.86] |
| Medication |  |  |
| Statins | 184 (49.7) | 431 (77.2) |
| Betablockers | 251 (67.8) | 490 (87.8) |
| ACE inhibitors | 85 (23.0) | 167 (29.9) |
| Diuretics | 88 (23.8) | 140 (25.1) |
