# Supplementary material for: Model selection for metabolomics: predicting diagnosis of coronary artery disease using automated machine learning
Source: Bioinformatics. 2019 Nov 8;36(6):1772–8. doi: 10.1093/bioinformatics/btz796 (PMC7703753; doi:10.1093/bioinformatics/btz796)
Supplement: btz796_Supplementary_Data [file btz796_supplementary_data.zip › btz796-Suppl_Data/TableS2.docx]

Table S2. Mean and standard deviation (SD) of 50 replicates of different TPOT configurations for P1 (A) and P2 (B)

| **Model** | **Mean (SD)** |
| --- | --- |
| 1. **P1** | |
| **TPOT** | ﻿0.72 (0.02) |
| **TPOT_LR** | 0.72 (0.02) |
| **TPOT_DT** | 0.61 (0.07) |
| **TPOT_RF** | 0.65 ( 0.05) |
| 1. **P2** | |
| **TPOT** | 0.74 (0.01) |
| **TPOT_LR** | 0.74 ( 0.02) |
| **TPOT_DT** | 0.72 ( 0.02) |
| **TPOT_RF** | 0.73 ( 0.02) |
